# Supplementary material for: Prevalence and associated factors of MAFLD in adults with type 2 diabetes
Source: PLOS Glob Public Health. 2024 Dec 30;4(12):e0003572. doi: 10.1371/journal.pgph.0003572 (PMC11684647; doi:10.1371/journal.pgph.0003572)
Supplement: Attachment — Submitted filename: renamed_e7c21.docx [file pgph.0003572.s001.docx]

Dear Editor-in-Chief,

Thank you very much for your letter and advice. We have revised the manuscript, and would like to re-submit it for your consideration. We have addressed the comments raised by editor and the reviewers, and the amendments are highlighted in the revised manuscript. Point by point responses to the reviewer’s comments are listed below this letter.

We hope that the revised version of the manuscript is now acceptable for publication in your journal.

I look forward to hearing from you soon.

With best wishes,

Yours sincerely,

Jin Lu

**Responses to Journal Requirements:**

Please amend your detailed Financial Disclosure statement. This is published with the article. It must therefore be completed in full sentences and contain the exact wording you wish to be published.

Reply : Thanks for your suggestion, we have modified it according to the requirements of the journal.

Please provide separate figure files in .tif or .eps format only and remove any figures embedded in your manuscript file. Please also ensure all files are under our size limit of 10MB.

Reply :We have removed the figure from the original text in accordance with the editor's request, and independently submitted the figure in .tif format as required by the journal.

We do not publish any copyright or trademark symbols that usually accompany proprietary names, eg  ©, ®, ™  (e.g. next to drug or reagent names). Please remove all instances of trademark/copyright symbols throughout the text, including ® on page

Reply : We have remove all instances of trademark/copyright symbols throughout the text.

**Responses to the reviewer’s comments**

We would like to express our sincere thanks to the reviewers for the constructive and positive comments.

Response to Reviewers

Reviewer #1: General comments:
• This cross-sectional study has very interesting findings for clinical practice and future studies. I congratulate the authors for their work on the predictors of steatosis and fibrosis amongst adult T2DM patients with MAFLD. The statistical analysis was great, the findings and discussions were well presented.
We thank the reviewer 1 for reading our paper carefully and giving the positive comments on our manuscripts. Your positive feedback and encouragement have been instrumental in enhancing the quality of the final manuscript. As for some weaknesses you mentioned, we have also made modifications one by one according to your suggestions.

• There are grammatical errors that need correction to ensure readability and clarity. The entire manuscript requires serious proofreading.

Reply :We have carefully checked the grammar and spelling of the whole article, and the revised part has been shown in the article.

o Page 5: under introduction, rephrase reference [1] by removing the ‘’.’’ Between syndrome and NAFLD and replace with a comma ‘’,’’. Also write NAFLD in full (Non-Alcoholic Fatty Liver Disease).

Reply :The sentence “With the epidemic of the globalization of obesity and metabolic syndrome. NAFLD has become the leading cause of chronic liver disease in developed regions[1] ”was modified to “ With the epidemic of the globalization of obesity and metabolic syndrome, NAFLD（Non-Alcoholic Fatty Liver Disease）has become the leading cause of chronic liver disease in developed regions[1] ”_。_
o Page 6: reference 6 should be rephrase e.g: To diagnose MAFLD, patients must first meet one or more of the following conditions: obesity or overweight, diabetes mellitus, metabolic disorders, which can be determined through blood biochemistry tests, anthropometric measurements, or medical history inquiries. Secondly, there must be evidence of steatosis or fibrosis in the liver. Liver biopsy remains the gold standard for diagnosis…….

Reply :Reference 6 has been modified as required.
o Page 7: include ‘’respectively’’ after 78% in reference 14 in relation to sensitivity and specificity.
Reply :The sentence was modified to “The sensitivity and specificity of these values are 69%, 77%, 88%, and 82%, 81%, 78% respectively. [14]^“^.

o Check: Alcoal(%)

Reply : I've modified the “Alcoal”
o Page 19, line 3: correct ‘’stuties’’ to ‘’studies’’ in relation to references 23 and 24 and also correct repetition after reference 27-29.

Reply:The requested modifications have been made as the requirements.
o Page 20, line 13 needs correction of the typo
Reply :Due to modifications in the discussion section, this literature has also been removed.
o There is no need to have ‘’ Funding None. Declaration of Competing Interest The authors declare that they have no known competing financial interests or personal relationships that could have appeared to influence the work reported in this paper.’’ After the conclusion since it is to be reflected in the matrix on page 1.

Reply :This content has been deleted.
• The lingering issue is the relationship between advanced fibrosis/cirrhosis and heart failure (causality or a consequence) and the pathogenesis/pathophysiology involved.

Reply:In the context of this topic, I have made revisions in the sixth paragraph of the discussion section. Firstly, I have analyzed their common pathogenic factors including chronic inflammatory state and RAAS activation. Additionally, both factors can also mutually promote each other. In the seventh paragraph, mechanisms promoting liver fibrosis in heart failure and promoting heart failure in liver fibrosis are separately analyzed.

Reviewer #2: The manuscript is technically deficient as the authors do not know what they are doing based on what is written in the abstract (logistic regression) and statistical analysis (linear regression). The weighted analysis is not described well. It also has many grammatical errors that could have been checked before submitting.

Reply :The description of weighted analysis has been re-added in the statistics section and marked in red. The entire text has been revised and polished.

Reviewer #3: The study to look in to: Prevalence and factors associated with MAFLD in adults with T2DM- the flow and presentation including justification, what is known and what the authors found is not clear. As it is a cross-sectional study, it is difficult to validate the findings presented as association between MAFLD and other factors. The association presented with increased prevalence of HF and Liver Fibrosis in adults with T2DM is not presented clearly to justify and validate the results.
It was not easy to read as the grammar and language including spellings need revision.

Reply :The flow chart has been redrawn, as shown in FIG. 1. In the discussion section, I have revised it according to the reasons why these data should be collected, the data results collected in this paper, the conclusions drawn by other authors, and the differences between our conclusions and those of other papers and the reasons. It is hoped that such changes can make reviewers and readers understand the issues to be explained in this article.

Since we are a cross-sectional study, it is true that causality cannot be determined as you said, so we have revised the wording describing the relationship between the two and changed the words such as “affect” to “relevant”.

Paragraphs 5, 6 and 7 of the discussion specifically describe the relationship between heart failure and fibrosis and their pathophysiological mechanisms. The entire text has been revised and polished. We hope the revised content can clearly explain the purpose, conclusion, and possible mechanisms of this manuscript.
