# Supplementary material for: Prevalence and associated factors of MAFLD in adults with type 2 diabetes
Source: PLOS Glob Public Health. 2024 Dec 30;4(12):e0003572. doi: 10.1371/journal.pgph.0003572 (PMC11684647; doi:10.1371/journal.pgph.0003572)
Supplement: Attachment — Submitted filename: renamed_fe228.docx [file pgph.0003572.s002.docx]

Dear Editor-in-Chief,

Thank you very much for your letter and advice. We have revised the manuscript, and would like to re-submit it for your consideration. We have addressed the comments raised by the reviewers, and the amendments are highlighted in the revised manuscript. Point by point responses to the reviewer’s comments are listed below this letter. During the revision process, we did not add or remove any citations.

We hope that the revised version of the manuscript is now acceptable for publication in your journal.

I look forward to hearing from you soon.

With best wishes,

Yours sincerely,

Jin Lu

**Responses to the reviewer’s comments**

We would like to express our sincere thanks to the reviewers for the constructive and positive comments.

Response to Reviewers

Reviewer #4: General comments:
Reviewer #4: The findings for the study are well represented and all issues raised by previous reviewers have been adequately addressed. However, the paper has some typos and minimal texts errors that need to be reviewed for clarity a simple proof reading should be able to resolve the issues. I have no further issues with the study.
Reply :We thank reviewer 4 for reading our paper carefully and providing positive comments on our manuscript. Your positive feedback and encouragement have been instrumental in enhancing the quality of the final manuscript. We have requested proofreading to identify any typographical or textual errors. Thank you for your valuable suggestions.

Reviewer #5: This is an interesting study and the authors are well appreciated for the idea and the statistical analysis. However, the manuscript still needs thorough revision.

1. The introduction section lacks clear idea of what the research problem and gap are .

Reply : In response to your feedback, we have incorporated the research questions and significance of this study into the introduction. Please refer to the modified sections highlighted in red within the introduction.

2. The objective of the study as mentioned, is not limited to Type 2 diabetes thus, the objective should include the association of MAFLD with variables other than Type 2 DM as observed in the statistical analysis and discussion section (eg. Hypertention, cardiac failure etc.)

Reply : As the flow chart shows, this study focused specifically on people with type 2 diabetes, rather than the general population. The objective of this study was to investigate the factors associated with fatty liver disease in individuals with type 2 diabetes mellitus, including demographic factors, laboratory indicators and several metabolic diseases.

3. The authors should clearly mention the nature of NHANES survey (cross-sectional or cohort) in the methodology section, as the authors try to establish a causal relationship of MAFLD with diabetes and other factors. If the survey was cross-sectional avoid making causal inferences. Also, justify including Liver function test parameters like ALT and AST as test variables. Raised ALT and AST are not the cause of MAFLD but an indicator of liver disease. There are several other variables whose inclusion into the study as a cause of MAFLD are not justified in the manuscript eg. GGT, Platelets count, HF, stroke etc.

Reply : We added "This is a cross-sectional study" to the methods section as you suggested. Readers may think that there are causation relationships due to the description of "risk factors" in the article；therefore we have changed "risk" to "associated" or "correlated". Some liver disease-related parameters such as ALT,AST, GGT, and PLT were added as predictors of statistics rather than causation. As stated in lines 331-332, "In addition, ALT, AST, and GGT were recognized as predictors of steatosis and fibrosis. "

The pathophysiological mechanisms underlying heart failure and stroke involve metabolic dysregulation. Therefore, we hypothesize that there is a relationship among heart failure, stroke, and one of the key components of metabolic syndrome, namely MAFLD. This hypothesis has been validated through statistical analysis. The exploration of causal relationships is discussed in detail in the discussion section.

4. The authors should justify that patients who were not classified as having Type 2 diabetes based on their HbA1c and fasting blood glucose levels were not actually cases of controlled diabetes.

Reply : We may have inaccurately described the inclusion and exclusion criteria. In fact, we have already accounted for the aforementioned population in our statistics. The original data indicate that some patients do not meet the diagnostic criteria for diabetes based on their glycosylated hemoglobin and blood glucose levels; however, they are still included in the statistical analysis (original data website：https://doi.org/10.5061/dryad.dz08kps5g). This group consists of individuals who have been diagnosed with diabetes but whose blood glucose levels are well-controlled. Therefore, we have added "absence of a history of T2DM" to the exclusion criteria in the Study Design and Methods section.

5. Please justify why individuals with type 1 DM were excluded from the study.

Reply : One of the requirements in the diagnostic criteria for MAFLD is type 2 diabetes rather than type 1 diabetes, and type 2 diabetes is more associated with metabolic disorders such as MAFLD, so type 1 diabetes is not included in this paper.

6. The discussion needs to be re-written to be in line with the objective of the study.

Reply : We have reorganized the order of the discussion section.Our objective was to investigate the prevalence of hepatic steatosis and liver fibrosis in individuals with type 2 diabetes, as well as the associated factors. All discussions are based on analyses conducted within this demographic; however, we have also compared our findings with data from the general population.

In the discussion section, the first paragraph addresses the prevalence rates, whereas the second paragraph focuses on factors related to hepatic steatosis. The third and fourth paragraphs examine factors associated with liver fibrosis. The fifth and sixth paragraphs emphasize the relationship between heart failure and MAFLD. The seventh paragraph discusses the limitations of our study, and the eighth paragraph provides a summary of our findings.

Given that numerous studies have already established associations between certain research objectives and MAFLD, this paper does not address those aspects in depth. However, heart failure has been identified as a disease associated with MAFLD in this study—a topic that is seldom mentioned in other literature; therefore, it receives particular emphasis in the discussion section.

7. Please avoid using the terms prevalence, or incidence in the results and discussion. The authors can rather recommend a randomized trial for causal inference based on the findings of the study.

Reply : We investigated the prevalence of MAFLD in a population with type 2 diabetes. Although there may be sampling errors, we incorporated the weights recommended by the database, which should provide a relatively accurate reflection of MAFLD prevalence in this population.We have revised the term "prevalence" to " weighted prevalence " . I changed the words " incidence rate of stroke” and “incidence rate of HF " to "occurrence of stroke " and “occurrence of HF”.

Thank you for the reminder about the necessity of a prospective design to elucidate causality. We also address this concern when discussing the limitations of our paper. For example, while only BMI is associated with fibrosis, blood glucose levels do not show a similar association; additionally, heart failure is linked to the progression of fibrosis.-+

7. There are numerous errors related grammatical and sentence structure. The whole manuscript needs english language expert editing.

Reply : This manuscript has been edited by native English speakers.
